# Complex Formation of an RNA Aptamer with a Part of HIV-1 Tat through Induction of Base Triples in Living Human Cells Proven by In-Cell NMR

**DOI:** 10.3390/ijms24109069

**Published:** 2023-05-22

**Authors:** Omar Eladl, Yudai Yamaoki, Keiko Kondo, Takashi Nagata, Masato Katahira

**Affiliations:** 1 Structural Energy Bioscience Research Section, Institute of Advanced Energy, Kyoto University, Kyoto 611-0011, Japan; omar.eladl.47r@st.kyoto-u.ac.jp (O.E.); yamaoki.yudai.7n@kyoto-u.ac.jp (Y.Y.); kondo.keiko.3u@kyoto-u.ac.jp (K.K.); nagata.takashi.6w@kyoto-u.ac.jp (T.N.); 2 Graduate School of Energy Science, Kyoto University, Kyoto 606-8501, Japan; 3 Faculty of Pharmacy, Zagazig University, Zagazig 44519, Egypt; 4 Integrated Research Center for Carbon Negative Science, Institute of Advanced Energy, Kyoto University, Uji 611-0011, Japan; 5 Biomass Product Tree Industry-Academia Collaborative Research Laboratory, Kyoto University, Kyoto 611-0011, Japan

**Keywords:** in-cell NMR, Tat, RNA aptamer, RNA–protein interaction, functional RNA, base triple

## Abstract

An RNA aptamer that strongly binds to a target molecule has the potential to be a nucleic acid drug inside living human cells. To investigate and improve this potential, it is critical to elucidate the structure and interaction of RNA aptamers inside living cells. We examined an RNA aptamer for HIV-1 Tat (TA), which had been found to trap Tat and repress its function in living human cells. We first used in vitro NMR to examine the interaction between TA and a part of Tat containing the binding site for trans-activation response element (TAR). It was revealed that two U-A∗U base triples are formed in TA upon binding of Tat. This was assumed to be critical for strong binding. Then, TA in complex with a part of Tat was incorporated into living human cells. The presence of two U-A∗U base triples was also revealed for the complex in living human cells by in-cell NMR. Thus, the activity of TA in living human cells was rationally elucidated by in-cell NMR.

## 1. Introduction

The inside of a living cell is extremely crowded with biological macromolecules. The structure, dynamics, and interactions of biomolecules could be different between in vitro and in-cell conditions. In-cell NMR spectroscopy is one of the most powerful techniques to address this point [1,2,3]. Therefore, investigation of these properties of RNA aptamers under in-cell conditions at atomic resolution is highly desired for making use of RNA aptamers as nucleic acid drugs. Toward this goal, previously, the in-cell NMR method has been utilized to investigate these properties in living human cells [4], including structures of a hairpin [5], i-motif [6,7], G-quadruplex [8,9], Z-DNA [10], and triplex [11], as well as the dynamics of hairpins and G-quadruplex [12]. Particularly, the in-cell NMR technique was applied to investigate the interactions between DNA and minor-groove binders [13], T-T mismatch and small compounds [13], G-quadruplex and small compounds [14], riboswitch and a ligand [15], RNA aptamer and a ligand [16], and mRNA and antisense nucleic acid analogs [17] in living human cells. The interactions between G-quadruplex and small compounds [18], and between riboswitch and a ligand [15], were also investigated in living *Xenopus laevis* (*X. laevis*) oocytes.

**Figure 1:**
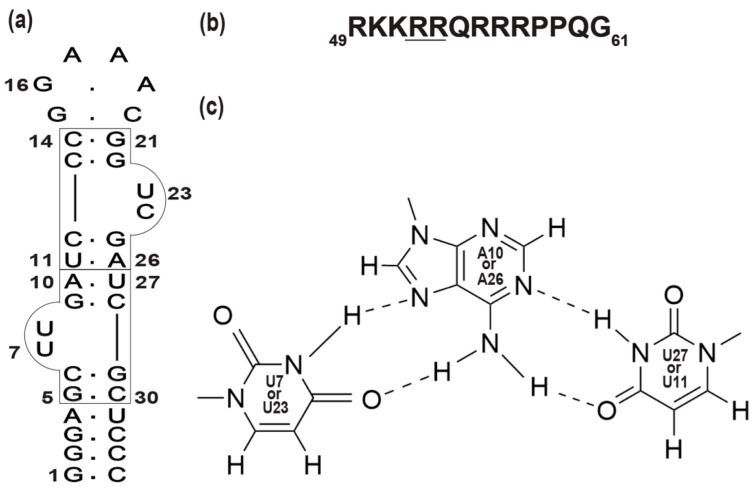
RNA aptamer for Tat (TA) and RG peptide: (**a**) The secondary structure of TA. The two adjacent Tat binding sites are boxed. The base pairs are indicated by dots. (**b**) The amino acid sequence of an arginine-rich peptide derived from the Tat protein (RG peptide). The critical arginine residues (R52 and R53) for the binding with TA [24] are underlined. (**c**) A schematic illustration of the U-A∗U base triples in TA.

In the replication cycle of human immunodeficiency virus-1 (HIV-1), the interaction between the trans-activator of transcription (Tat) protein and trans-activation response element (TAR) located in a transcript of viral long terminal repeats has a crucial regulatory role in the elongation during HIV transcription [19,20,21]. Previously, we succeeded in identifying a novel RNA aptamer for HIV-1 Tat (TA) (Figure 1a) with two TAR-like motifs [22,23]. TA specifically bound to Tat with over 100 times higher binding affinity than the authentic TAR RNA, and the aptamer effectively inhibited the Tat-dependent trans-activation of transcription in the nuclear extract and the living cells, acting as a decoy for Tat [22,23].

The structural features responsible for the extremely high affinity of TA for Tat were studied by NMR under in vitro conditions. The two TAR-like binding boxes (Figure 1a) enable TA to interact with Tat at double binding sites simultaneously through the formation of two adjacent U-A∗U base triples: U7*A10-U27 and U23*A26-U11 (Figure 1c). The formation of U-A∗U base triples results in the wider major groove that is required for the access of arginine residues of Tat (Figure 1b) to the binding sites G9 and G25 (Figure 1a) [22,23,24]. However, there have been no structural studies under in-cell conditions; thus, the origin of the high binding affinity of TA to Tat in living human cells is elusive.

In our previous study, we succeeded in detecting the interaction between the related RNA aptamer for HIV-1 Tat (TA_36 that has extra UC residues at the 3′-end of TA) and argininamide—which is the simplest analog of the HIV-1 Tat protein in living human cells—by using in-cell NMR [16]. The in-cell NMR spectra suggested that the structure of the complex of TA_36 with argininamide under in-cell conditions is similar to that under in vitro conditions [16]. However, the critical structural features of the RNA aptamer (either TA_36 or TA)—the formation of U-A∗U base triples—have not been proven in living human cells. Here, to elucidate the mechanism of the high binding affinity of the RNA aptamer to Tat in living human cells, we extended the in-cell NMR application to investigation of the interaction of RNA with a part of Tat that is a more realistic target molecule than argininamide. In the present study, we performed in-cell NMR of TA with the arginine–rich region of Tat (RG peptide, _49_RKKRRQRRRPPQG_61_), and we investigated the structure and interaction of the TA-RG peptide complex in living human cells.

## 2. Results

### 2.1. Complex Formation of TA with the RG Peptide, with the Explicit Evidence of U-A∗U Base Triples

**Figure 2:**
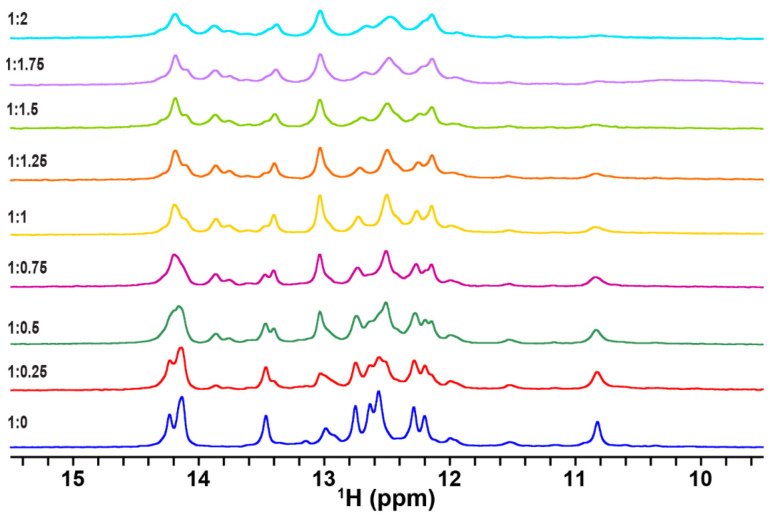
In vitro ^1^H NMR spectra of TA (150 μM) at different molar ratios of [TA]:[RG peptide] in transport buffer (TB) at 10 °C. The imino proton region is shown.

Firstly, to determine characteristic features of the NMR spectra of TA in free and complex forms with the RG peptide, NMR spectra of TA were recorded at different molar ratios of [TA]:[RG peptide]. Upon binding of TA with the RG peptide, new imino proton signals appeared (Figure 2), indicating that the binding process is in a slow exchange regime on the NMR timescale. The spectral change reached a plateau at the molar ratio of [TA]:[RG peptide] = 1:1 (Figure 2). This indicates that the binding stoichiometry of the TA-RG peptide complex is 1:1.

**Figure 3:**
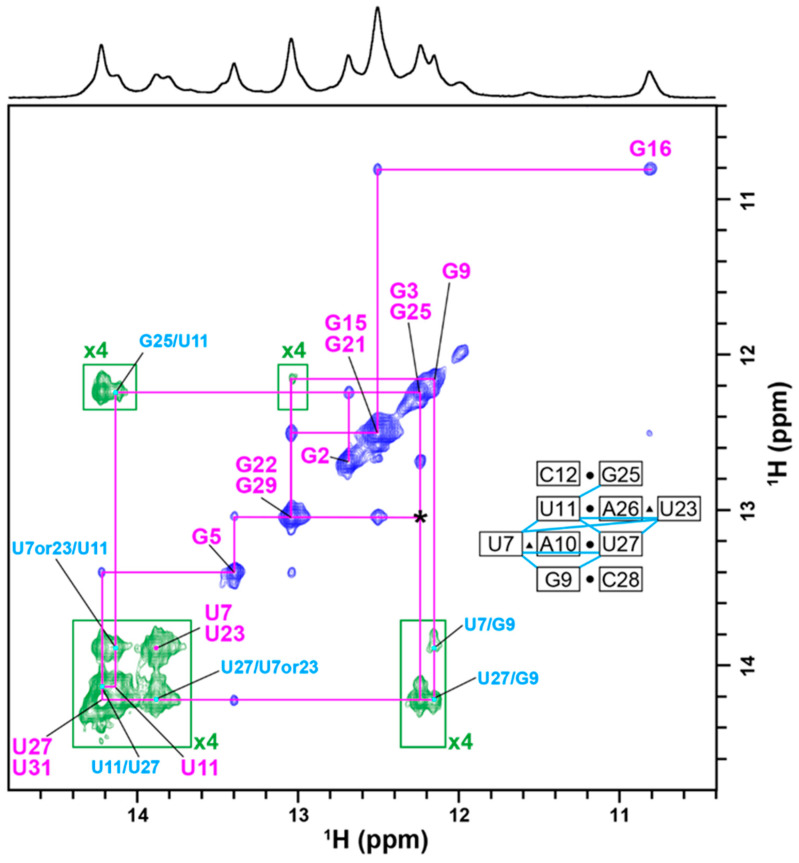
Assignments of resonances: In vitro ^1^H-^1^H NOESY spectrum (imino proton region) of the TA-RG peptide complex (1:1) at 5 °C and pH 7.0. The assignments are indicated with lines, and the residue numbers in magenta. Signals whose intensities are multiplied by four are indicated in green. The asterisk indicates a signal that is visible at lower contour levels. The inter-residue NOEs with the uracil residues involved in the two base triples are labeled in light blue. The inset is a schematic representation of the binding site of TA. The observed NOEs are indicated as light blue lines.

There were two newly appearing imino proton signals at 13.75 and 13.85 ppm. These two signals were not observed when arginine (Figure S1) or argininamide (Figure S2)—the simplest analogs of the Tat peptide—was bound to TA. To identify the origins of these two newly appearing imino proton signals of TA upon binding of the RG peptide, a 2D ^1^H-^1^H NOESY spectrum of the TA-RG peptide complex was recorded, and signal assignments were carried out (Figure 3). The imino proton signals of TA in a complex with equimolar RG peptide were sequentially assigned, as shown in Figure 3. The obtained assignments were consistent with those of TA in a complex with two argininamides [22]. The two newly appearing signals at 13.75 and 13.85 ppm were assigned to the imino proton of either U7 or U23 of the Hoogsteen-type A*U base pairs involved in the U-A∗U base triples. These assignments were supported by many NOEs observed for two U-A∗U base triples (U7*A10-U27 and U23*A26-U11) and two adjacent G-C base pairs (G9-C28 and C12-G25) indicated in an inset of Figure 3. The appearance of imino proton signals of U7 and U23 could be used to examine the structure and mode of interaction of the TA-RG peptide complex in living human cells, because these signals do not appear if TA interacts with arginine (Figure S1), which is abundantly present in living human cells.

Previously, we investigated the TA-RG peptide complex by NMR [22]. The imino proton signals of U7 and U23 were not observed in that case. This was assumed to be due to the lack of magnesium ions in that case. In fact, the imino proton signals of U7 and U23 were not observable in magnesium-ion-excluded TB (Figure S3). Furthermore, the melting temperature (*T*_m_) of the TA-RG peptide complexes increased from 60.1 °C in the absence of magnesium ions to over 75 °C in their presence (Figure S4). These results confirmed that we could observe those signals thanks to the presence of 2.5 mM magnesium ions that stabilized the TA-RG peptide complex, including U-A∗U base triples, probably through weakening of the repulsion between negatively charged phosphate groups [25].

### 2.2. Intermolecular NOEs between TA and the RG Peptide

**Figure 4:**
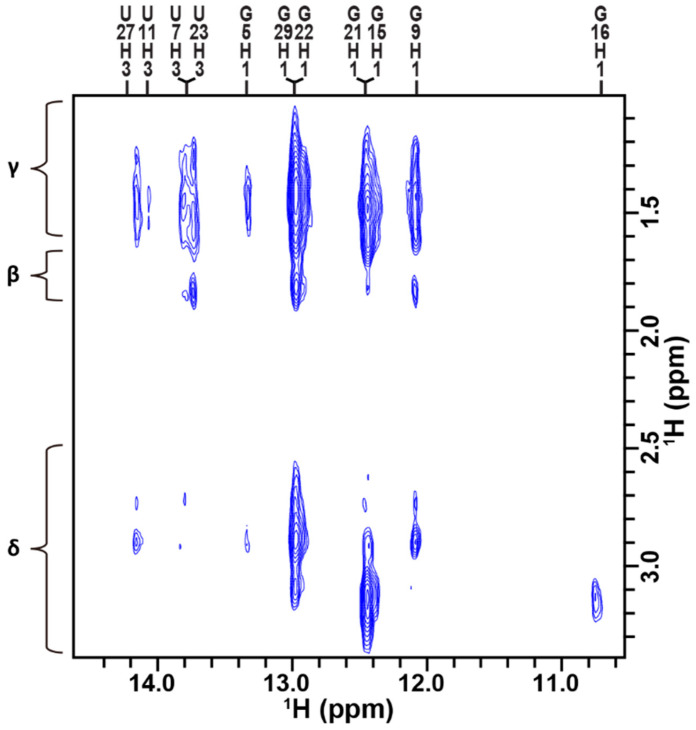
Intermolecular NOEs between TA and the RG peptide. In vitro NOESY cross-peaks between the imino protons of TA and Hβ/Hγ/Hδ of arginine residues of the RG peptide observed in TB at 5 °C.

Many intermolecular NOEs were observed between the imino proton resonances of TA and the Hβ/Hγ/Hδ resonances of Arg, Lys, Pro, and Gln residues of the RG peptide (Figure 4). This is a direct indication of the complex formation. The intermolecular NOE cross-peaks between TA and the RG peptide were analyzed.

The intermolecular NOEs observed for G9, U11, G22, G29, and U27 of TA were assumed to be those with critical Arg residues—R52 and R53 (see also the legend of Figure 1)—of the RG peptide, since the corresponding intermolecular NOEs were observed for the TA:arginineamide complex [22]. Those intermolecular NOEs were also observed in our previous study on the TA:RG peptide complex [22]. As explained in the introduction, G9 and G25 are binding sites. The five residues mentioned above—G9, U11, G22, G29, and U27—are all located at or next to binding sites. Therefore, it is reasonable to observe intermolecular NOEs with critical Arg residues for those residues of the aptamer.

The intermolecular NOEs observed for G5, G15, G16, and G21 may involve those with not only Arg but also Lys, Pro, and Gln, since the corresponding intermolecular NOEs were not observed for the TA:arginineamide complex [22]. Those four residues are separate from the binding sites. Therefore, it is not likely to observe intermolecular NOEs with critical Arg residues for those four residues of the aptamer. In that sense, the observation of intermolecular NOEs with Lys, Pro, and Gln residues is likely for these four residues.

It should be noted that intermolecular NOEs were observed for U7 and U23. These intermolecular NOEs were not observed for the TA:arginineamide complex, because imino proton resonances were absent for these residues. The intermolecular NOEs observed for U7 and U23 of the TA:RG peptide complex might be with the critical Arg residues, because these residues of the aptamer are located next to the binding sites G9 and G25.

### 2.3. Confocal Fluorescence Microscopy Analysis

**Figure 5:**
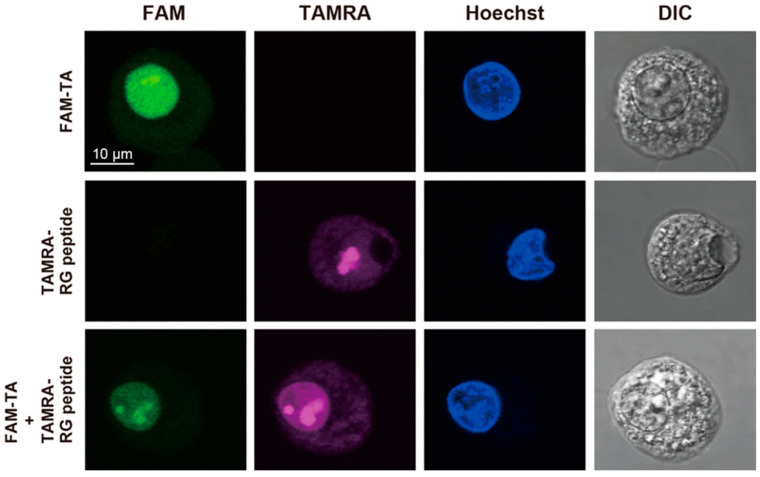
Intracellular distribution of FAM-labeled TA and TAMRA-labeled RG peptide. Confocal fluorescence microscopy images of HeLa cells treated by electroporation with FAM-labeled TA alone (**top**), TAMRA-labeled RG peptide alone (**middle**), and the mixture of FAM-labeled TA and TAMRA-labeled RG peptide (**bottom**). The cell nuclei were stained with Hoechst 33342. Differential interference contrast (DIC) images are shown in the fourth column. Bar = 10 μm.

Here, we examined the localization of FAM-labeled TA and TAMRA-labeled RG peptide for three individual samples by confocal fluorescence microscopy. For the first two samples, FAM-labeled TA alone and TAMRA-labeled RG peptide alone were introduced into HeLa cells (Figure 5). For the third sample, FAM-labeled TA and TAMRA-labeled RG peptide were introduced as a complex into HeLa cells. In all samples, electroporation was used to introduce the RNA and peptide into the cells.

In Figure 5 (top), the RNA-alone images indicate that FAM-TA is mostly, if not all, present in the nucleus, i.e., localized in the nucleus; many bright dots were observed. In Figure 5 (middle), the peptide-alone images indicate that TAMRA-RG is distributed both in the nucleus and cytosol; there were regions brighter than the other regions inside the nucleus. In Figure 5 (bottom) and Figure S5, the RNA–peptide complex images indicate that FAM-TA is mostly, if not all, present in the nucleus, i.e., localized in the nucleus, while TAMRA-RG is distributed in both the nucleus and the cytosol. TAMRA-RG was brighter in the nucleus than in the cytosol. Additionally, there were regions brighter than the other regions inside the nucleus for the fluorescence intensities of FAM-TA and TAMRA-RG; these brighter regions for FAM-TA and TAMRA-RG were superimposed very well. This observation may indicate the presence of the FAM-TA:TAMRA-RG complex.

It is hard to completely exclude the possibility that both the peptide and RNA could localize by themselves in the nucleus. Therefore, we confirmed the presence of the complex by in-cell NMR, where we observed the imino proton signals for U-A∗U base triples, which is direct evidence for the presence of the complex.

### 2.4. In-Cell NMR Spectrum of TA in Complex with the RG Peptide in Living Human Cells

**Figure 6:**
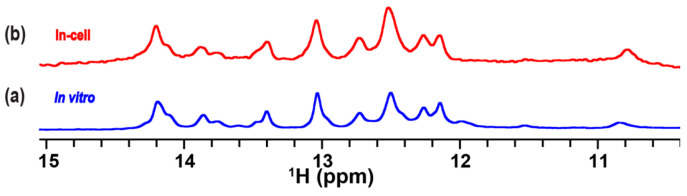
In-cell ^1^H NMR spectra of TA: Imino proton spectrum of TA in a complex with the RG peptide recorded in transport buffer (TB) under in vitro (**a**) and in-cell conditions (**b**).

The TA-RG peptide complex was incorporated into HeLa cells, and a 1D ^1^H in-cell NMR spectrum was recorded. The imino proton signals of TA inside the cells were observed (Figure 6b). The spectral pattern of the in-cell NMR spectrum was quite similar to that of the in vitro spectrum of TA in a complex with the RG peptide (Figure 6a). Particularly, the imino proton signals at 13.75 and 13.85 ppm that corresponded to the presence of U7*A10-U27 and U23*A26-U11 base triples, respectively, were observed. These results indicate that the TA-RG peptide complex is formed with two U-A∗U base triples under in-cell conditions.

## 3. Discussion

In this study, our main intention was to expand the application of the in-cell NMR technique for investigation of the interaction between functional RNA and its binding partner inside cells. To prove this concept, we needed to use a well-characterized complex comprising an RNA and its specific binding partner, which has high affinity. This is why we intentionally chose an RNA aptamer for Tat (TA) and the arginine-rich region of Tat (RG peptide), which we had characterized previously.

We detected the presence of a complex of the RNA aptamer TA with the RG peptide derived from the HIV-1 Tat protein in living human cells by in-cell NMR (Figure 6). Previously, we and another group demonstrated the interactions between an RNA and a small compound in living human cells using in-cell NMR [15,16]. Here, we performed the measurement of an in-cell NMR spectrum of the RNA aptamer with its target peptide—RG peptide—for the first time. This study expands the field of application of the in-cell NMR technique for investigation of the interaction behavior of a functional RNA—a more complicated system to be studied.

As elucidated in our previous study on the complex under in vitro conditions, the formation of two U-A∗U base triples widens the originally narrow major groove and, thus, produces space for the accommodation of two key arginine residues of Tat—R52 and R53—for binding [22,24]. This is the origin of the high affinity of TA toward Tat. Here, we demonstrated that the TA-RG peptide complex is present with the induction of two U-A∗U base triples under in-cell conditions as well.

This can rationalize the high affinity of TA toward Tat observed under in-cell conditions [23]. In fact, it was revealed that TA can trap Tat and, thus, effectively repress the elongation of the transcription aided by Tat in HeLa cells [23].

The in-cell NMR spectrum of TA showed that TA forms a complex with the RG peptide even in living human cells, where large amounts of endogenous proteins, nucleic acids, and small molecules (such as metabolites) are present. This suggests that the interaction of TA with the RG peptide is highly specific and was not inhibited by the endogenous molecules—particularly those such as arginines and basic proteins. This specific interaction is essential for the development of a therapeutic molecule that functions as a decoy for Tat and inhibits the elongation of HIV transcription in living human cells.

## 4. Materials and Methods

### 4.1. Preparation of RNA and RG Peptide

An RNA aptamer for HIV-1 Tat (TA, 5′-GGGAGCUUGAUCCCGGAAACGGUCGAUCGCUCCC-3′) was synthesized, purified, and desalted by Hokkaido System Science Co., Ltd. (Hokkaido, Japan). Tat-derived arginine-rich peptide (RG peptide, _49_RKKRRQRRRPPQG_61_) was synthesized and purified by Toray Research Center, Inc. (Tokyo, Japan). The concentrations of TA and RG peptide were measured based on the UV absorbance at 260 and 214 nm, respectively. The molecular extinction coefficient of RG peptide at 214 nm was deduced as described in a previous report [26].

### 4.2. In Vitro NMR Measurements

The in vitro NMR experiment on either TA alone or with the RG peptide was performed in transport buffer (TB: 25 mM HEPES-KOH (pH 7), 115 mM CH_3_COOK, 2.5 mM MgCl_2_) containing 5% D_2_O and 10 μM 4,4-dimethyl-4-silapentane-1-sulfonic acid (DSS). The RNA solution was annealed at 90 °C for 5 min and then slowly cooled to 4 °C at a rate of −1 °C/min using a thermal cycler (TaKaRa RCR Thermal cycle Dice Gradient) (Takara, Otsu, Japan). In vitro NMR spectra were recorded using the Band-Selective Optimized-Flip-Angle Short-Transient (SOFAST) [27] technique with PC9 and rSNOB pulses centered at 12.5 ppm, covering a bandwidth of 5.0 ppm. The number of scans for 1D ^1^H NMR spectra was 512. A 2D NOESY spectrum was recorded with 192 scans and 2048 (t2) × 512 (t1) complex points. The mixing time for NOESY was 300 msec. The direct ^1^H dimension for both 1D and 2D spectra covered only the imino proton chemical shift regions. All in vitro 1D and 2D spectra were recorded at 10 °C and 5 °C, respectively, using a Bruker BioSpin AVANCE III HD 600 spectrometer equipped with a cryogenic probe. The concentrations were 150 μM and 1 mM for the 1D and 2D experiments, respectively. All NMR spectra were processed and analyzed using Bruker TopSpin 3.6.4 and Poky (ver20220114) software [28].

### 4.3. In-Cell NMR Measurements Using a Bioreactor System

TA annealed with the RG peptide was introduced into HeLa cells by the electroporation method [6,15,29] using an NEPA21 Super Electroporator system (Nepa Gene Co., Ltd., Chiba, Japan), as described previously [16]. Prior to electroporation, HeLa cells were harvested and washed with phosphate-buffered saline (PBS). The cells were resuspended in 450 μL of TB containing 1 mM TA annealed with 2 mM RG peptide. The total 600 μL of the cell suspension was divided into three electroporation cuvettes (2 mm gap). The cuvettes were incubated on ice for 10 min, and then electroporation was conducted with two poring pulses (125 V) separated by 50 msec intervals, and sequentially followed by five transferring pulses (15 V) separated by 50 msec intervals. Then, immediately, the electroporated cells were transferred to 20 mL of prewarmed DMEM and allowed to recover for 15 min at 37 °C.

The cells were centrifuged (300× *g*, 5min, 4 °C), and the supernatant was removed. Then, the cell pellet was washed with 10 mL of 0.9 × Leibovitz’s L-15 medium containing 10% D_2_O, and 20 μM DSS, and the supernatant was removed by centrifugation. This washing process was repeated twice. Then, the cells were centrifuged (300× *g*, 5min, 4 °C), and an 80% slurry of the electroporated cells was prepared by resuspending the cells in 0.9 × Leibovitz’s L-15 medium containing 10% D_2_O and 20 μM DSS. A mixture of 40% cells and 1.5% agarose was prepared by mixing the same volume of the 80% cell slurry with 3% low-melting Seaprep agarose (Lonza, Basel, Switzerland) in 0.9 × Leibovitz’s L-15 medium containing 10% D_2_O and 20 μM DSS. The resulting mixture was transferred to a 0.5 mm inner diameter polytetrafluoroethylene (PTFE) tube and then incubated on ice for 10 min. The encapsulated cells in crumpled agarose gel threads were transferred to a 5 mm NMR tube with 140 μL of 3% agarose gel in 0.9 × L-15 medium containing 10% D_2_O and 20 μM DSS at its bottom. On the crumpled agarose gel threads with cells, 140 μL of 1.5% crumpled agarose gel thread without cells that had been solidified in a 1 mm inner diameter silicon tube was added. Finally, the NMR tube was manually centrifuged to form packed agarose gel thread layers.

The setup of the bioreactor system for in-cell NMR [30,31] was as described previously [11]. In the bioreactor system, fresh medium was continuously supplied to the NMR tube during the in-cell NMR measurements to extend the lifetime of the cells. Fresh 0.9 × Leibovitz’s L-15 medium containing 10% D_2_O and 20 μM DSS was transferred to the NMR tube with a syringe pump at a flow rate of 25 μL/min, through an inlet PTFE tube that was connected to the NMR tube. The exhausted medium was drained from the NMR tube to a waste bottle through an outlet PTFE tube.

The in-cell NMR spectrum was recorded at 10 °C using the SOFAST technique in the same manner as the in vitro NMR spectra. The number of scans was 8192, and the total acquisition time was 2 h.

### 4.4. Confocal Fluorescence Microscopy Analysis

Here, 2.5 × 10^6^ HeLa cells were resuspended in 150 μL of TB containing 10 µM FAM-labeled TA alone, 10 µM TAMRA-labeled RG peptide alone, or the complex of FAM-labeled TA with TAMRA-labeled RG peptide (1:1). The total volume of each cell suspension was 200 μL. The cells treated by electroporation were resuspended in the L-15 medium containing 10 µM Hoechst 33342, and then incubated for 20 min in a 35 mm polylysine-coated glass-bottomed dish (Iwaki, Chiba, Japan) at room temperature. Fluorescence microscope images were acquired with an Olympus FV1000 confocal scanning laser microscope equipped with a 60 × UPlanSApo objective.

### 4.5. Temperature-Dependent UV Spectroscopy

The melting analysis on 5 μM TA-RG peptide complex (1:1) was carried out in either TB or magnesium-ion-excluded TB. The UV absorbance at 280 nm [32] was recorded using a JASCO V-750 spectrophotometer equipped with a Peltier temperature-control device. The sample temperature was ramped at 0.5 °C/min from 15 to 85 °C. The UV melting curves were analyzed by taking the first derivative of the absorbance with respect to temperature, as described in a previous report [32,33].

## 5. Conclusions

In conclusion, we obtained the first in-cell NMR spectrum of an RNA aptamer in a complex with a peptide. The in-cell NMR spectrum showed that the structure of the TA-RG peptide complex under in-cell conditions is similar to that under in vitro conditions. Particularly, it was revealed that the two U-A∗U base triples that are essential for the high binding affinity of TA to Tat were formed in living human cells. These findings enable us to understand the origin of the reported high binding affinity of TA for Tat in living human cells. Our present study demonstrates that in-cell NMR spectroscopy can be used to assess the structure of an RNA aptamer in a complex with its target peptide. The structural insight as to an RNA aptamer in living human cells is essential for understanding of the origin of the high binding affinity of the RNA aptamer inside the cells, as well as for developing RNA aptamer-based drugs.

## Data Availability

Not applicable.

## Supplementary Materials

The following supporting information can be downloaded at: https://www.mdpi.com/article/10.3390/ijms24109069/s1.
