# Supplementary material for: Complex Formation of an RNA Aptamer with a Part of HIV-1 Tat through Induction of Base Triples in Living Human Cells Proven by In-Cell NMR
Source: Int J Mol Sci. 2023 May 22;24(10):9069. doi: 10.3390/ijms24109069 (PMC10218998; doi:10.3390/ijms24109069)
Supplement: Supplementary file 1 [file ijms-24-09069-s001.zip › ijms-2359246-supplementary.pdf]

International Journal of *Molecular Sciences*

MDPI

Complex formation of an RNA aptamer with a part of HIV-1 Tat through induction of base triples in living human cells proved by in-cell NMR

Omar Eladl [1,2,3,†], Yudai Yamaoki [1,2,4,†], Keiko Kondo [1,4,5], Takashi Nagata [1,2,4] and Masato Katahira [1,2,4,5,*]

1 Structural Energy Bioscience Research Section, Institute of Advanced Energy, Kyoto University, Kyoto 611-0011, Japan; omar.eladl.47r@st.kyoto-u.ac.jp (O.E.); yamaoki.yudai.7n@kyoto-u.ac.jp (Y.Y.); kondo.keiko.3u@kyoto-u.ac.jp (K.K.); nagata.takashi.6w@kyoto-u.ac.jp (T.N.)
2 Graduate School of Energy Science, Kyoto University, Kyoto 606-8501, Japan
3 Faculty of Pharmacy, Zagazig University, Zagazig 44519, Egypt
4 Integrated Research Center for Carbon Negative Science, Institute of Advanced Energy, Kyoto University, Uji 611-0011, Japan
5 Biomass Product Tree Industry-Academia Collaborative Research Laboratory, Kyoto University, Kyoto 611-0011, Japan
* Correspondence: katahira.masato.6u@kyoto-u.ac.jp
† These authors contributed equally to this work.

Supporting information

Figure S1. *In vitro* ^{1}H NMR spectra of TA (150 μM) at different molar ratios of [TA]:[Arginine] in transport buffer (TB) at 10°C. Imino proton region is shown.

Figure S2. *In vitro* ^{1}H NMR spectra of TA (150 μM) at different molar ratios of [TA]:[Argininamide] in transport buffer (TB) at 10°C. Imino proton region is shown.

Figure S3. *In vitro* ^{1}H NMR spectra of TA (150 μM) at different molar ratios of [TA]:[RG peptide] in magnesium ion-excluded TB at 10°C. Imino proton region is shown.

Figure S4. UV-melting curves for the TA-RG peptide complex. The melting curve for the TA-RG peptide complex in TB (orange) and magnesium ion-excluded TB (black).

Figure S5. Intracellular distribution of FAM-labeled TA and TAMRA-labeled RG peptide. Confocal fluorescence microscopy images of HeLa cells treated by electroporation with the complex of FAM-labeled TA and TAMRA-labeled RG peptide. The cell nuclei were stained with Hoechst 33342. Fluorescence images for FAM-labeled TA (a), TAMRA-labeled RG peptide (b), and Hoechst 33342 (c). Bar = 30 μm.

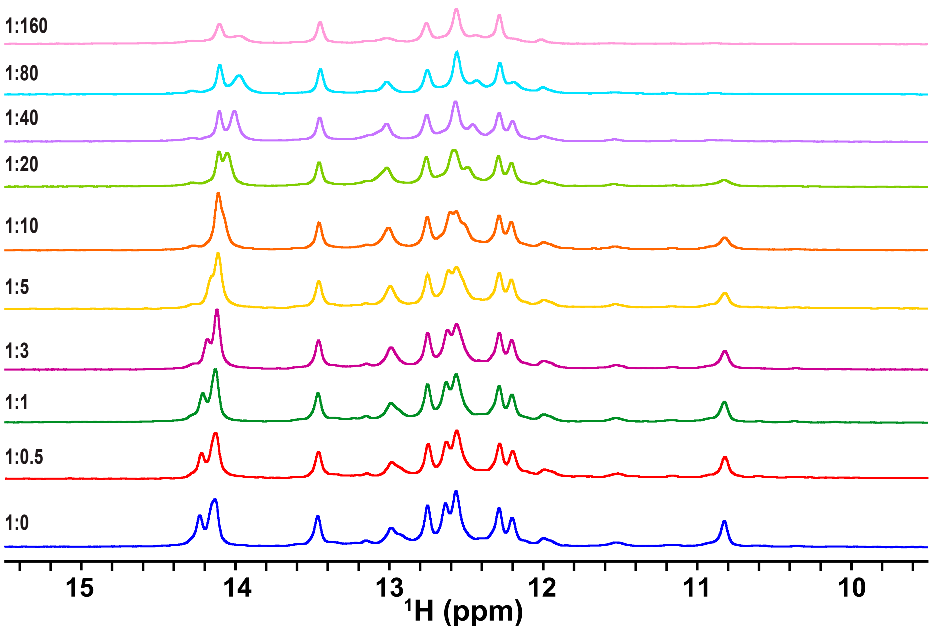

Figure S1. *In vitro* ^{1}H NMR spectra of TA (150 μM) at different molar ratios of [TA]:[Arginine] in transport buffer (TB) at 10°C. Imino proton region is shown.

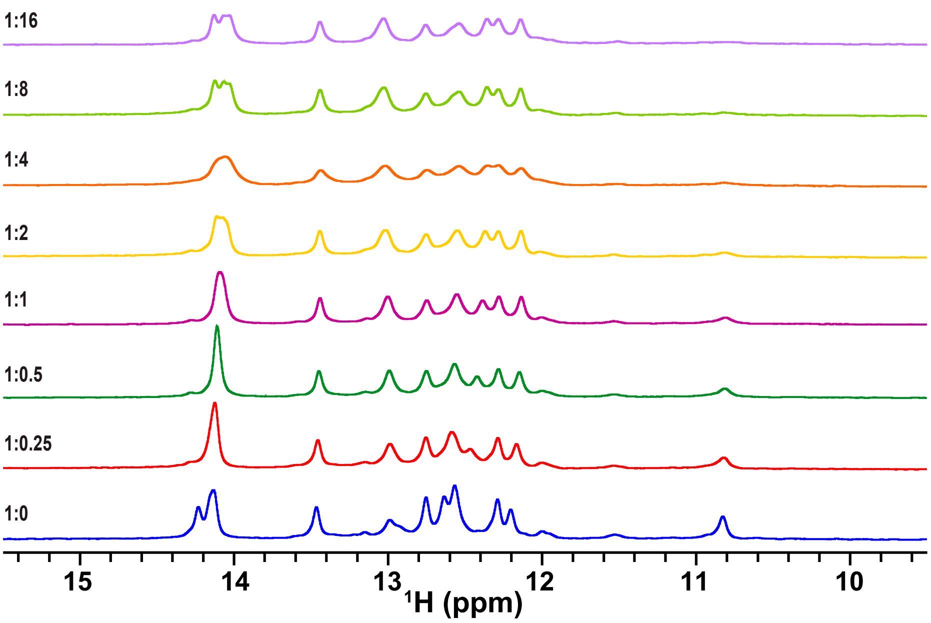

Figure S2. *In vitro* ^{1}H NMR spectra of TA (150 μM) at different molar ratios of [TA]:[Argininamide] in transport buffer (TB) at 10°C. Imino proton region is shown.

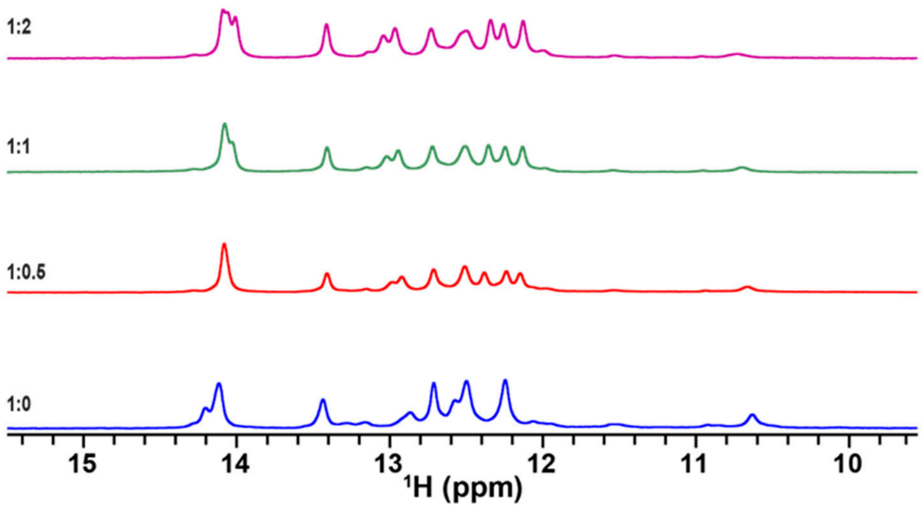

Figure S3. *In vitro* ^{1}H NMR spectra of TA (150 μM) at different molar ratios of [TA]:[RG peptide] in magnesium ion-excluded TB at 10°C. Imino proton region is shown.

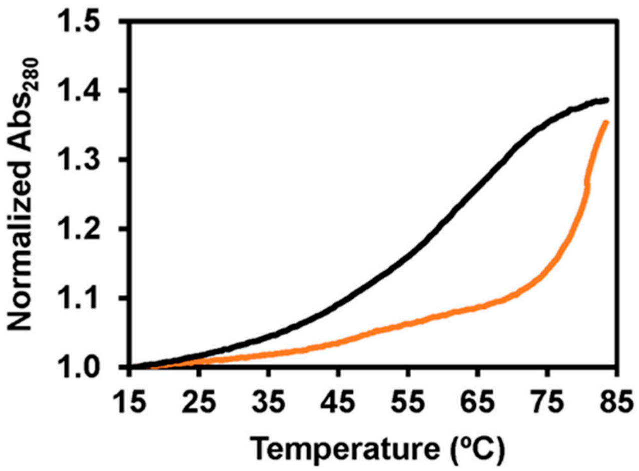

Figure S4. UV-melting curves for the TA-RG peptide complex. The melting curve for the TA-RG peptide complex in TB (orange) and magnesium ion-excluded TB (black).

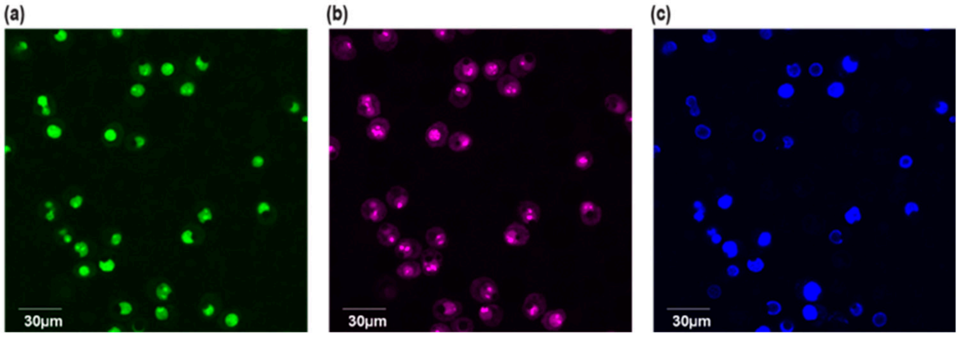

Figure S5. Intracellular distribution of FAM-labeled TA and TAMRA-labeled RG peptide. Confocal fluorescence microscopy images of HeLa cells treated by electroporation with the complex of FAM-labeled TA and TAMRA-labeled RG peptide. The cell nuclei were stained with Hoechst 33342. Fluorescence images for FAM-labeled TA (a), TAMRA-labeled RG peptide (b), and Hoechst 33342 (c). Bar = 30 μm.
